# Optical Strategy Utilizing Contrast Modulation to Slow Myopia

**DOI:** 10.1016/j.xops.2024.100672

**Published:** 2024-12-09

**Authors:** James S. Wolffsohn, Kate L. Gifford

**Affiliations:** 1 School of Optometry, College of Health and Life Sciences, Aston University, Birmingham, UK; 2 Optometry and Vision Science, Queensland University of Technology, Brisbane, Australia; 3 Myopia Profile Pty Ltd, Brisbane, Australia

**Keywords:** Contrast modulation, Myopia control, Diffusion optics, ON–OFF pathways

## Abstract

A new method to slow myopia progression utilizes Diffusion Optics Technology (DOT) spectacle lenses. The proposed mechanism of action for the DOT lenses is to modulate contrast across the photoreceptor cells, leading to an altered activity of the ON and OFF pathways and slowing the progression of axial elongation. This approach is different from the current optical approaches that utilize optical defocus to reduce hyperopic defocus at the peripheral retina although central vision is fully corrected to slow myopia. Initial clinical studies with the DOT lenses have demonstrated promising results with a reduction in progression of myopia. This overview summarizes the current knowledge on myopia risk factors, the evidence for involvement of contrast signaling pathways in refractive error development, and the theories and mechanisms behind DOT lens technology. It also considers the role of contrast and the paradoxical observations given the established paradigm of form deprivation in animal models.

**Financial Disclosure(s):**

Proprietary or commercial disclosure may be found in the Footnotes and Disclosures at the end of this article.

The human eye has sophisticated mechanisms that respond, adjust, and adapt to visual signals to enable sharpness across a wide range of environments. For instance, consider the manner in which vision is maintained daily across dynamic environments with varying levels of luminance and contrast such as indoor to outdoor settings, overcast to bright conditions, or from mid-day blue to sunset red hue of the sky. Given the versatility of the eye to respond and adapt to such complex temporal and spatial conditions, the development and progression of refractive errors is puzzling. Of the refractive errors, myopia is of significance due to its fast-rising global prevalence and the substantial health and economic burden it imposes on individuals and societies.^1^ Estimated to affect approximately 50% of the world’s population by the year 2050,^2^ it is already an epidemic in many East Asian countries where children as young as 3 to 4 years have myopia,^3^ >80% of the young adult population is myopic, and a significant number of individuals have myopia over −6.00 diopters (D).^4^ With each diopter increase in myopia said to be associated with a 58%, 20%, 21%, and 30% risk in myopic maculopathy, open-angle glaucoma, cataract, and retinal detachment, respectively,^5^ the data forebode a future public health crisis.

Given the burden of myopia, the argument for the use of strategies to prevent or slow progression is compelling.^6^^,^^7^ Modeling the reduced risk of retinal pathologies if myopia was reduced using multiple approaches indicates significant benefits with adoption of myopia control strategies. A strategy that can potentially slow myopia progression by even −1.0 D can significantly lower the number of years spent with visual impairment and decrease the risk of developing myopia-related retinal complications.^5^ Among myopia management approaches, spectacles are a practical option for children. Additionally, compared with standard single-vision spectacles, which do not slow the progression of myopia, the reduced progression from myopia-controlling strategies offers benefits of better unaided vision, improved productivity, and reduced risk of future vision impairment and complications.^8^

On a positive note, there already exist environmental factors such as time outdoors and optical, pharmaceutical, and light-based strategies to slow the progression of myopia.^9^, ^10^, ^11^ So far, strategies underpinning optical approaches have mostly considered “defocus blur” with hyperopic defocus at either, or, the central and peripheral retina as the predominant mechanism underlying development and progression of myopia.^9^ A new, alternate strategy termed Diffusion Optics Technology (DOT) utilizes light scattering centers in the peripheral treatment zone to modulate or dampen “abnormal contrast signaling” at the photoreceptor mosaic in the peripheral retina and, consequently, slow axial elongation. Early results from human clinical trials with DOT spectacle lenses indicate successful control of myopia progression in children as young as 6 years old.^12^ The concept is thought-provoking given the use of light scattering for contrast modulation rather than defocus blur to slow myopia and the paradoxical observation *vis-à-vis* form-deprivation myopia. Hence, it is timely to review the current understanding of the risk factors for myopia, consider the role of contrast in refractive error development, and explore the mechanisms for slowing myopia with DOT lenses compared to other strategies.

## Risk Factors for Myopia

Many distinct risk factors have been associated with myopia and include younger age, Asian ethnicity, parental myopia, female sex, disrupted sleep cycle, increased near work, reduced outdoor time, education, socioeconomic status, urban living, intelligence, and peripheral refractive error asymmetry.^13^ The strength of association for each of these many risk factors with myopia is difficult to delineate due to confounders; however, it is argued that the evidence is conclusive in isolation for (1) increased education and (2) reduced time outdoors being causal risk factors.^13^ Although genetic factors do play a role in onset and progression, the fast-rising prevalence of myopia is considered indicative of the greater potency of the environmental risk factors over genetic factors.^13^

**Figure 1:**
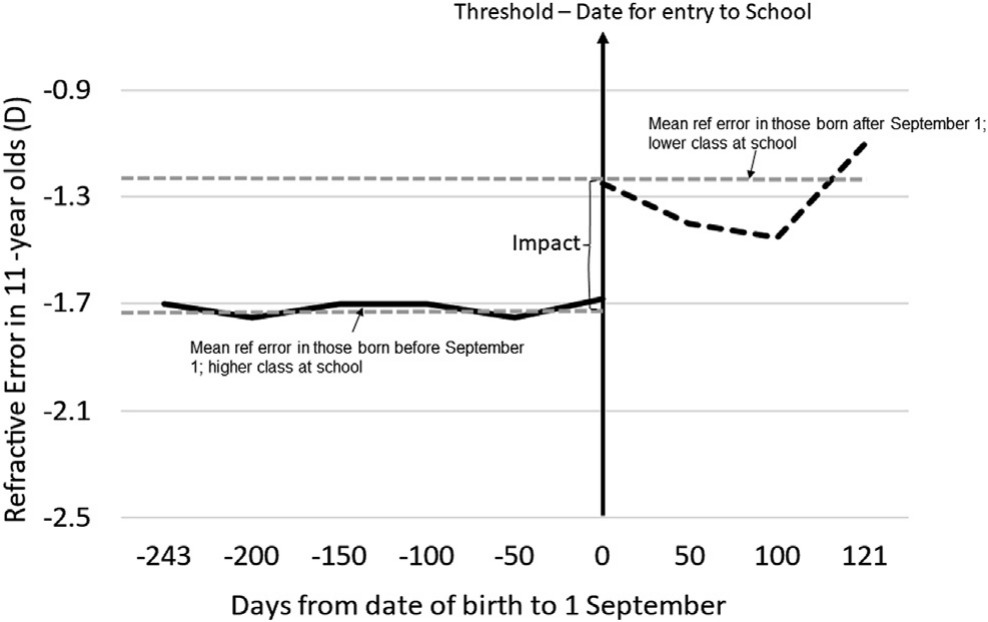
Regression discontinuity analysis illustrating the effect of age cutoff criteria for school entry on refractive error in urban China. Adapted from He et al.^14^ Children born before September 1 are in a higher class and have a more myopic refractive error compared with those born after September 1 and in a lower class at school. D = diopters.

Regression discontinuity analyses conducted using large samples indicate the impact that education has on myopia; at any specific age, children who have a higher academic load have a higher risk of developing myopia (Fig 1).^14^ The behavioral aspects or features that link the educational environment to myopia are not entirely clear; however, near-based activities intrinsic to modern educational settings that entail prolonged viewing of high-contrast stimuli with many hours spent on near work, continuous near work without breaks, at much lower lighting intensity and of a different spectrum than that are found outdoors, and reading and writing at close distances are frequently associated with myopia.^15^, ^16^, ^17^ Normal text types involve high-contrast targets, and their role in myopia has been studied before. There is contrast adaptation during a reading text task in emmetropes and myopes; however, myopic eyes show greater adaptation.^18^^,^^19^ Contrast adaptation leads to altered sensitivity and is considered to play a role in myopia,^19^ although it could also result from myopic ocular changes. Furthermore, recent observations indicate that children in lower socioeconomic/migrant schools may be at increased risk due to possibly being in spaces with inadequate light, limited outdoor time, and facilities.^20^, ^21^, ^22^

The protective effect of more time outdoors on preventing myopia is well established,^11^ but the mechanisms that provide this benefit are not well understood. Some of the factors thought to play a role include brightness of light, spectral composition of outdoor light, a uniform dioptric field with reduced hyperopic defocus for outdoor and distant targets, smaller pupil size resulting in increased depth of focus, and reduced accommodative effort.^23^ In a large-scale observational study involving children wearing light sensors over a year, exposure to higher light intensity was associated with reduced incidence of myopia.^24^ Myopes also tended to spend less time exposed to bright light (>5000 lux),^17^ and high/bright light was found to inhibit form-deprivation myopia in animal models.^25^ The spatial frequency composition of indoor and outdoor urban environments has also been proposed as a contributor to myopia when compared to natural environments, described as a ratio of contrast across spatial frequencies.^26^ The indoor environment examined was shown to consist of less high spatial frequencies than the outdoors, but the spatial frequencies at the retina depend on the accommodative state of the eye and visual field covered by the object of regard; thus, for emmetropic eyes, the peripheral retina is most often filled with gentle, low-contrast images of distant, out-of-focus scenery that do not drive axial elongation, according to contrast theory.

There are still many questions about these risk factors and their relationship with myopia; however, it seems that individuals who spend a significant amount of time indoors engaged in near-based, high-contrast activities with less exposure to outdoors and bright light are at increased risk.

## Contrast and Optical Defocus in Refractive Error Development

It has long been considered that eye growth and refractive error development are modulated by visual feedback.^27^ The exact mechanism remains to be elucidated, but defocus blur is considered to play an important role and is backed by multiple lines of evidence.^28^ Defocus or blur occurs when the focal plane is formed either in front of (myopic defocus) or behind (hyperopic defocus) the retina. However, only optical defocus creates a focal point in front of or behind the retina; therefore, it is considered a closed-loop condition. Low-pass filtering of an image, by decreasing contrast across spatial frequencies, creates a blur that does not have a focal point; therefore, it presents an open-loop condition. It can be caused by the mismatch between the optical power of the eye and its eye length or can be imposed artificially with optical lenses. Imposed optical defocus accurately modulates growth in eyes across a range of animal species, imposed positive optical defocus results in eye shortening, and negative optical defocus results in eye lengthening that matches the imposed defocus.^29^ With the removal of imposed defocus, the eye loses the anatomical changes acquired in response to defocus, recovers, and returns to a state as observed in control untreated eyes.^30^ Furthermore, there is a large and growing body of literature from human clinical trials supporting slowing of myopia with peripheral optical defocus.^9^^,^^10^^,^^31^ The mechanisms regulating eye growth were demonstrated in animal models to be local; hemiretinal and local deprivations induced local changes.^28^^,^^29^ Additionally, despite lesioning/sectioning of the Edinger–Westphal nucleus, the ciliary ganglion, or the optic nerve, the eye continued to compensate for the imposed defocus.^32^, ^33^, ^34^

However, optical defocus blur alone does not fully explain certain observations. If optical defocus fine-tunes the eye to grow toward emmetropia, the reason for a myopic eye to continue to grow despite having previously attained emmetropia remains unclear. Despite the convincing evidence from animal studies indicating compensation for myopic defocus, in human trials, undercorrection failed to slow myopia.^35^^,^^36^ Furthermore, progressive addition lenses or bifocals that impose myopic defocus across large sections of the retina are less efficient in slowing myopia compared with the multisegment-type spectacle and contact lenses.^9^ Additionally, if the eye is sensitive to defocus and responds by matching the eye length to the imposed defocus, the reason for the myopic eye to demonstrate better tolerance to optical defocus and adaptation to blur is unclear.^37^^,^^38^ These observations suggest the possibility of other interrelated higher-order processing pathways involving contrast in emmetropization and refractive error development. In chick eyes, a strong effect on eye growth was observed when contrast was significantly reduced while other properties, such as luminance and spatial frequency, were held constant;^39^ this is the opposite effect found with mild adjustment of contrast with DOT lenses in humans.^12^ Many species use contrast signaling ON and OFF pathways to differentiate light falling at the retina into light and dark stimuli and process them in an independent and parallel manner.^29^^,^^40^ It is useful to briefly consider the role of these pathways in refractive error development.

Contrast is the difference in luminance and color of an object from its surroundings that makes it distinguishable. In the eye, the channels that encode contrast are well established and include a vast network of photoreceptor cells, bipolar cells, amacrine cells, horizontal cells, and ganglion cells at the retina that are organized into separate receptive fields known as ON and OFF pathways.^41^ When the retinal photoreceptor cells detect and respond to the presence of light, the information is relayed to bipolar cells that are organized as ON (respond to light or positive contrast) and OFF (respond to absence or dimming of light or negative contrast) cells. The mechanisms involved in the pathway are extensively researched and can be reviewed in detail elsewhere.^42^

Data from both animal and human studies demonstrate associations between one or more retinal cells that signal contrast and the ON–OFF pathways in emmetropization and development of refractive errors.^40^^,^^43^ In human eyes, myopia is a significant feature of eyes with ON bipolar cell dysfunctions, cone and rod dystrophies.^44^^,^^45^ Of these, especially the bipolar cells and photoreceptors were considered critical for myopia development; both cone photoreceptor and ON bipolar dysfunctions are associated with high levels of myopia.^44^ Disturbances at different levels of the ON–OFF pathway are considered to explain the variants in congenital stationary night blindness; with incomplete congenital stationary night blindness, both ON and OFF responses were attenuated, whereas in complete congenital stationary night blindness, only the ON response was attenuated.^46^

In experimental animal models, nonfunctioning ON pathways were found to be involved with more myopic shifts, but no change in dopamine levels,^47^ whereas nonfunctional OFF pathways did not have much influence on myopia, although they had increased dopamine levels,^48^ leading to the conclusion that ON pathway transmission is more important.^43^^,^^49^ For example, blocking ON pathways in eyes of kittens with intravitreal injections of D,L-2-amino-4-phosphonobutyric acid resulted in hyperopia.^50^ However, in other experiments, interfering with the ON OFF pathways influenced and varied the refractive error, but the pattern and the involvement of either the ON or OFF pathway were not always consistent. For example, in recent studies involving chicks, although dynamic ON stimuli resulted in choroidal thickening and OFF stimuli resulted in choroidal thinning, both paradigms resulted in more myopia.^43^ In an earlier experiment, chick eyes exposed to a temporal, low-contrast sawtooth profile target with a fast ON response failed to compensate to impose hyperopic defocus (negative lenses) and instead became relatively hyperopic.^51^

These results suggest that perturbations in one or more cells or levels of the contrast signaling pathways might be involved in refractive error development including myopia. It should be noted that the rules for processing these signals and the involvement of any particular cell type are not yet well understood. Furthermore, the path from phototransduction to influencing eye growth remains to be clarified.

## Can Contrast Modulation Be Used to Slow Myopia Progression?

There is evidence that connects environmental risk factors for myopia to ON–OFF pathways; reading dark text on light background (thought to stimulate the OFF pathway) resulted in choroidal thinning.^43^^,^^52^^,^^53^ Conversely, bright text on dark background resulted in choroidal thickening,^43^ but later studies failed to replicate this finding.^53^ In individuals with longer axial lengths, there was reduced sensitivity to light than dark targets, suggesting a decreased sensitivity to the ON pathway.^54^ Visual environments such as optical blur and low light are thought to weaken ON response and promote myopia progression.^55^ Reading and viewing high-contrast targets promoted contrast adaptation,^19^ and at high contrast, dark stimuli were located faster with a domination of the OFF pathway.^56^^,^^57^

The development of DOT for slowing myopia by modulating contrast is said to have originated from observations of syndromic high myopia. In Bornholm eye disease, a familial form of high myopia, the genetic locus myopia 1 X-linked is located on the X chromosome at Xq28, where the long-wavelength and middle-wavelength cone opsin genes reside. Certain rare versions of these opsin gene haplotypes, notably LVAVA and LIAVA, were directly linked to syndromic and nonsyndromic high myopia that maps to myopia 1 X-linked. They demonstrated significant exon-3 skipping leading to deficit of the opsin (photopigment) in affected (mutant) cones.^58^, ^59^, ^60^, ^61^ The intermixing of mutant and normal cones across the photoreceptor mosaic produces a high-contrast differential between adjacent cones, leading to an abnormal activation of both ON and OFF pathways despite the absence of stimuli; the consequence of the excessive activity in the contrast pathways is an increased eye elongation.^61^ Even low to moderate myopia is associated with cone opsin gene polymorphism that occurs with high frequency in the population producing a contrast differential between adjacent cones (as in Bornholm eye disease, but much smaller).^62^^,^^63^ Additionally, viewing high-contrast scenes can lead to elevated activity of both the ON and OFF pathways, for example, when reading black text on white paper, which may be considered sources of man-made contrast. Using DOT lens technology to modulate the contrast is thought to reduce activation^64^ of the excessive firing of the contrast signaling pathways^40^^,^^64^^,^^65^ and thus slow eye elongation.^12^

In a large-scale multicenter clinical trial in North America involving 256 children with myopia, progression of myopia was compared between 2 test spectacle lenses comprising DOT and single-vision spectacles. The purpose of the applied diffusive microdots was to scatter light and hence reduce contrast across a large range of spatial frequencies without significantly compromising visual acuity, therefore resulting in slower axial elongation. Test lens 1 referred to as DOT 0.2 had fewer diffusive microdots, whereas test lens 2 differed by having a higher density of microdots. Both the test lenses incorporated a base power that corrected for the refractive error of the eye and further incorporated diffusive microdots across the lens except for a clear central zone. After 1 year, wear of both test lenses resulted in slowed progression of myopia compared with wear of single-vision spectacles. Slower progression was observed with test 1 (50% or 0.15 mm reduction in axial elongation and 74% or 0.40 D reduction in spherical equivalent) compared with test 2 (33% or 0.10 mm reduction in axial elongation and 50% or 0.32 D reduction in spherical equivalent).^12^ The lack of evidence for a dose–response effect—with test lens 2 having a higher density of microdots but a lower efficacy for myopia control—was likely related to a higher volume of dropouts and compliance issues.^12^ Specifically, 41% of children wearing test lens 2 reported removing the spectacles for near activities, compared with <20% in test 1 and control lenses. For test lens 1, a larger absolute treatment effect was observed in the younger children aged 6 to 7 years (n = 78), where refractive progression was 74% or 0.56 D (0.22 mm change in axial length) slower in test 1 and 56% or 0.42 D (0.21 mm change in axial length) slower in test 2 groups compared with the control group.^12^

## Diffusion Optics, Other Myopia Control Optical Strategies, Atropine, and Form Deprivation

Table 1 illustrates the proposed mechanism of action for current myopia control optical strategies utilizing defocus versus DOT lenses and provides a comparison with form deprivation models. While the current optical strategies utilize optical power or defocus blur to shift the focal plane and reduce the hyperopic defocus at the central and, or, peripheral retina, the DOT lens technology utilizes translucent microdots to scatter and reduce contrast signaling at the retina.

**Table 1:** Comparison of Myopia Control Strategies Utilizing Defocus Blur vs. Diffusion Optics

	Optical Defocus for Myopia Control	Diffusion Optics	Form Deprivation
Lens design	Lens incorporates base power to correct for the distance refractive error Relatively positive powered lens segments/regions are located on the lens inferiorly (e.g., bifocal, progressive addition lenses),^9^ peripherally, or in the center of the lens (e.g., peripheral hyperopia reducing spectacles, multifocal contact lenses, multisegment spectacles)^9^^,^^66^, ^67^, ^68^ extended depth of focus ( EDOF) contact lens is an exception where the power profile incorporates both relatively positive and negative regions^69^	Lens incorporates base power to correct for the distance refractive error Translucent diffusive microdots scattered/positioned across lens with clear spaces between the microdots^12^	No refractive error correction-translucent diffuser or Bangerter foils of varying strengths mounted on rings and attached to front of eyes
Assessed in	Children with myopia: bifocals, progressive additional lenses, multifocal/multisegment spectacles and contact lenses, and orthokeratology lenses Experimental animal models	Children with myopia	Experimental animal models Nonmyopic eyes were reported in ocular conditions such as congenital ptosis and congenital cataract
Proposed mechanism	In addition to correcting for the refractive error of the eye, relatively positive powered regions reduce hyperopic defocus and, or, impose myopic defocus at the retina → ↓ axial elongation	Diffuse regions scatter light to reduce contrast→ minimizes contrast differential at retinal photoreceptors →decreased firing of neuronal ON–OFF pathways → ↓ axial elongation	Deprivation of form (pattern) → ↑ axial elongation (open loop)
Outcome	High contrast visual acuity mostly unaffected^70^^,^^71^ Reduced axial elongation Varied efficacy depending on lens type Compliance improves efficacy^72^^,^^73^	High-contrast visual acuity mostly unaffected^74^ Reduced axial elongation Both higher and lower density slow myopia Increased compliance leads to better outcome	Reduction in visual acuity^75^ Excessive axial elongation in animal models Graded phenomenon: Higher strength diffusers result in higher levels of myopia. Low strength induces minimal to nil myopia
Schematic			

Provided is a comparison of form deprivation models. → = leads to; ↓ = decreased.

Contrast modulation theory is not related to enhanced or impaired contrast sensitivity; however, if the DOT lens technology works by modulating contrast, it is likely that it attenuates and, or, modifies the intensity of the contrast signal at one or more frequencies, which would result in altered or decreased contrast sensitivity. It was reported that contrast sensitivity was not significantly reduced when viewing through the central clear aperture or the treatment zone of DOT lenses.^76^ Continuing this reasoning, it raises a query as to whether the existing myopia control approaches also involve contrast modulation. With multifocal or multizone contact lenses and multisegment spectacle lens designs used to slow myopia progression, high-contrast visual acuity remains mostly unaffected, but contrast sensitivity is altered or reduced when viewing through the treatment portion.^69^, ^70^, ^71^^,^^77^, ^78^, ^79^ Interestingly, no decrement in contrast sensitivity was observed with atropine 0.01% in a short-term study.^80^ This finding is not surprising given that 0.01% atropine has minimal effect on pupil size, accommodative response, or axial elongation,^81^ and it needs to be determined if more effective formulations and, or, higher concentrations affect contrast sensitivity.

Animal research has established that signals derived from both contrast and defocus can influence refractive development. In the retina, the pathways that encode myopic and hyperopic defocus are unique and different from those that process contrast signals.^82^ In form-deprivation myopia, a well-established paradigm replicated across many animal species, use of translucent, frosted lenses or Bangerter filters that filter out pattern or detail from viewing scenes results in axial elongation and subsequent myopic refractive error.^29^^,^^83^ Indeed, in monkeys, even peripheral form deprivation disrupted emmetropization, with the majority having relative levels of myopia.^84^ On this basis, it seems counterintuitive that DOT lenses slow myopia given the diffusion zone fills most of the spectacle lens. However, comparison of the results with DOT versus form deprivation from animal models indicates distinct differences. Firstly, form deprivation was found to be a graded phenomenon; diffusers of higher strength showed a significant decrease in visual acuity and contrast sensitivity accompanied by a significant myopic shift. The lower-strength diffusers also affected visual acuity and contrast sensitivity but resulted in either minimal myopia or no difference in refractive error compared with control eyes.^47^^,^^85^ In comparison, DOT lenses were made with a clear center and a peripheral treatment area designed to mildly reduce contrast, likely providing a different visual experience than such diffusers. Unlike form deprivation, where even low-strength diffusers resulted in some development of myopia, wear of both the lower- and higher-density DOT lenses slowed myopia progression. Moreover, the evidence for form-deprivation myopia in human eyes with congenital ptosis and cataract is inconclusive and does not seem to follow the classical animal model for form deprivation; compared with an earlier case review,^86^ in recent studies involving 30 and 37 patients with congenital ptosis, respectively, axial length and prevalence of myopia were not different between the ptotic and fellow eyes.^87^^,^^88^

It is evident that long-term follow-up and additional observations with DOT lenses are needed to confirm the promising initial results and further explore their mechanism of action. Although it is puzzling that the higher gradation lenses showed lower myopia control efficacy—likely due to wearability issues leading to higher dropout rate and potentially poorer wearer compliance, affecting sample size—the data need to be examined further. Nevertheless, it seems that reduced contrast acts to slow myopia progression in human eyes in the unique paradigm and intervention provided by DOT lenses.

## Summary

Spectacle lenses comprising DOT technology are considered to slow myopia by modulating or reducing contrast signaling to manage myopia. The approach is different to the existing optical strategies that use defocus to influence and slow progression of myopia. There is some evidence for involvement of contrast signaling pathways in emmetropization and refractive error development, but this requires further exploration. The pathways that encode contrast might also be involved in encoding defocus and thus might be interrelated. Although the use of DOT lenses seems counterintuitive given our current understanding of the influence of blur and form deprivation on myopia development in animal models, examination of the evidence indicates significant differences in the DOT lens approach and application to the human visual system. Further information on long-term efficacy will provide better understanding of the technology as compared to other strategies used to slow myopia.
